# Systematic review of phase‐I/II trials enrolling refractory and recurrent Ewing sarcoma: Actual knowledge and future directions to optimize the research

**DOI:** 10.1002/cam4.3712

**Published:** 2021-01-15

**Authors:** Arthur Felix, Pablo Berlanga, Maud Toulmonde, Judith Landman‐Parker, Sarah Dumont, Gilles Vassal, Marie‐Cécile Le Deley, Nathalie Gaspar

**Affiliations:** ^1^ Department of Oncology for Child and Adolescent Gustave Roussy Cancer Campus Villejuif cedex France; ^2^ Medical Oncology Department Institut Bergonié Bordeaux France; ^3^ Service d'Hématologie et d'Oncologie Pédiatrique Hôpital Armand Trousseau Paris France; ^4^ Department of Medical Oncology Gustave Roussy Cancer Campus Villejuif France; ^5^ Direction de la Recherche Clinique et de l'Innovation Centre Oscar Lambret Lille France

**Keywords:** Ewing sarcoma, new cancer therapies, phase‐I/II trials, trial design

## Abstract

**Background:**

Optimal Phase‐II design to evaluate new therapies in refractory/relapsed Ewing sarcomas (ES) remains imperfectly defined.

**Objectives:**

Recurrent/refractory ES phase‐I/II trials analysis to improve trials design.

**Methods:**

Comprehensive review of therapeutic trials registered on five databases (who.int/trialsearch, clinicaltrials.gov, clinicaltrialsregister.eu, e‐cancer.fr, and umin.ac.jp) and/or published in PubMed/ASCO/ESMO websites, between 2005 and 2018, using the criterion: (Ewing sarcoma OR bone sarcoma OR sarcoma) AND (Phase‐I or Phase‐II).

**Results:**

The 146 trials identified (77 phase‐I/II, 67 phase‐II, and 2 phase‐II/III) tested targeted (34%), chemo‐ (23%), immune therapies (19%), or combined therapies (24%). Twenty‐three trials were ES specific and 48 had a specific ES stratum. Usually multicentric (88%), few trials were international (30%). Inclusion criteria cover the recurrent ES age range for only 12% of trials and allowed only accrual of measurable diseases (RECIST criteria). Single‐arm design was the most frequent (88%) testing mainly single drugs (61%), only 5% were randomized. Primary efficacy outcome was response rate (RR=CR+PR; Complete+Partial response) (n = 116/146; 79%), rarely progression‐free or overall survival (16% PFS and 3% OS). H0 and H1 hypotheses were variable (3%–25% and 20%–50%, respectively). The 62 published trials enrolled 827 ES patients. RR was poor (10%; 15 CR=1.7%, 68 PR=8.3%). Stable disease was the best response for 186 patients (25%). Median PFS/OS was of 1.9 (range 1.3–14.7) and 7.6 months (5–30), respectively. Eleven (18%) published trials were considered positive, with median RR/PFS/OS of 15% (7%–30%), 4.5 (1.3–10), and 16.6 months (6.9–30), respectively.

**Conclusion:**

This review supports the need to develop the international randomized phase‐II trials across all age ranges with PFS as primary endpoint.

## Graphical abstract

Hundred and forty‐six phase‐II trials and 62 articles from 2005 to 2018 were analysed. Mostly multicentric, few trials were international (30%), only 5% were randomized. Results of 62 published trials enrolling 827 ES patients were disappointing. Combining chemotherapy with another agent is a promising strategy. International randomized trans‐age with PFS as primary endpoint could be promoted.

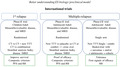

## 1 INTRODUCTION

Ewing sarcoma (ES), characterized by a specific transcript,^1^ represents the second most frequent bone cancer in adolescents and young adults (incidence 1–3 cases/million people/year).^1^ This rare cancer occurs at a median age at diagnosis of 14 years old, with 20% of patients older than 20 years.

Since the 1970 s survival of localized ES patients (70% 5‐year‐OS) has significantly improved, through international collaborative phase‐III trials regardless of patients’ age.^2^, ^3^ However, survival improvement was null in newly diagnosed multi‐metastatic ES and in refractory/recurrent ES (OS<20% at 5 years) despite aggressive multimodality treatments.^4^, ^5^ Thus, new anti‐ES drugs are urgently needed. Despite the large number of new drugs explored in the last 15 years, none has been yet successfully routinely implemented in first‐line or second‐line recurrent ES treatment. With increasing costs of clinical trials, Phase‐II studies must be designed to allow accurate interpretation of the results and further development of a drug in phase‐III trials. However, there is no specific recommendation for the design and reporting of phase II trials in oncology.

The aim of our study was to review the literature regarding phase‐II efficacy trials conducted in patients with recurrent/refractory ES between 2005 and 2018, according to PRISMA methodology,^6^ analyze, and expose encountered issues to help optimizing the design of next generation trials and drug development strategies.

## 2 METHODS

### 2.1 Search strategy and selection criteria

Systematic search for phase‐I/II clinical therapeutic trials in recurrent/refractory ES opened to recruitment between 01/01/2005 and 31/08/2018. Initial search was: (Ewing sarcoma OR bone sarcoma) AND (Phase 2 OR Phase‐II) on five international clinical trial registries: International Clinical Trials Registry Platform (ICTRP) of the World Health Organization (who.int/trialsearch, WHO), United States National Library of Medicine (ClinicalTrials.gov, NCT), European Clinical Trials Database (EMA), French National Cancer Institute (INCa) registry, and University hospital Medical Information Network (UMIN) for medical schools in Japan. The search was enlarged to (Ewing sarcoma OR bone sarcoma OR sarcoma) AND (Phase 2 OR Phase‐II OR Phase 1 OR Phase‐I) on ClinicalTrials.gov, and who.int/trialsearch.

Then, related articles/abstracts were searched on PubMed, the American Society of Clinical Oncology (ASCO), and European Society for Medical Oncology (ESMO) websites. To ensure exhaustiveness, we also performed a search on these websites using (Ewing sarcoma OR sarcoma) AND (Phase 2 OR Phase‐II) AND (“2005”–“2020”) to recover undeclared missing trials.

The last search was performed in April 2020. Two authors performed the trial eligibility assessment independently in non‐blinded standardized manner. Disagreements were resolved by consensus with a third author.

### 2.2 Data extraction

Data extraction was performed by two authors according to a standardized data extraction sheet (Table S1), using all available publications as long as specific results for ES stratum were shown. In case of multiple reports from the same trial, the report with the longest follow‐up was used. Disagreements were resolved by discussion with a third author.

For multistage designs, we collected the total planned sample size, even if trial was ongoing.

The therapeutic interventions were classified in categories based on mechanisms of action: 1) Targeted therapy (small molecules, excluding monoclonal antibodies) alone or combined, 2) Immunotherapy (antibodies, lymphocytes, cytokines, and viruses) alone or combined, 3) Chemotherapy single drug or combined, 4) Treatment involving radiotherapy, and 5) Combined therapies (chemotherapy+immunotherapy, chemotherapy+targeted therapy, targeted therapy+immunotherapy, combination of the 3).

### 2.3 Statistics

The list of trials matching eligibility criteria was merged using R software (3.2). The response rates (RRs) and disease control rates (DCR) were calculated/extrapolated from the publications, for every trial with 10 or more ES patients accrued, from the number of patients achieving complete response (CR), partial response (PR) (RR=CR+PR), or disease stabilization (SD) (DCR=CR+PR+SD). Phase‐II trials were considered positive if efficacy results reached the main outcome as defined per each protocol. They were considered negative if efficacy results did not reach the main outcome as defined per each protocol.

## 3 RESULTS

### 3.1 Eligible trials and information extraction

**FIGURE 1:**
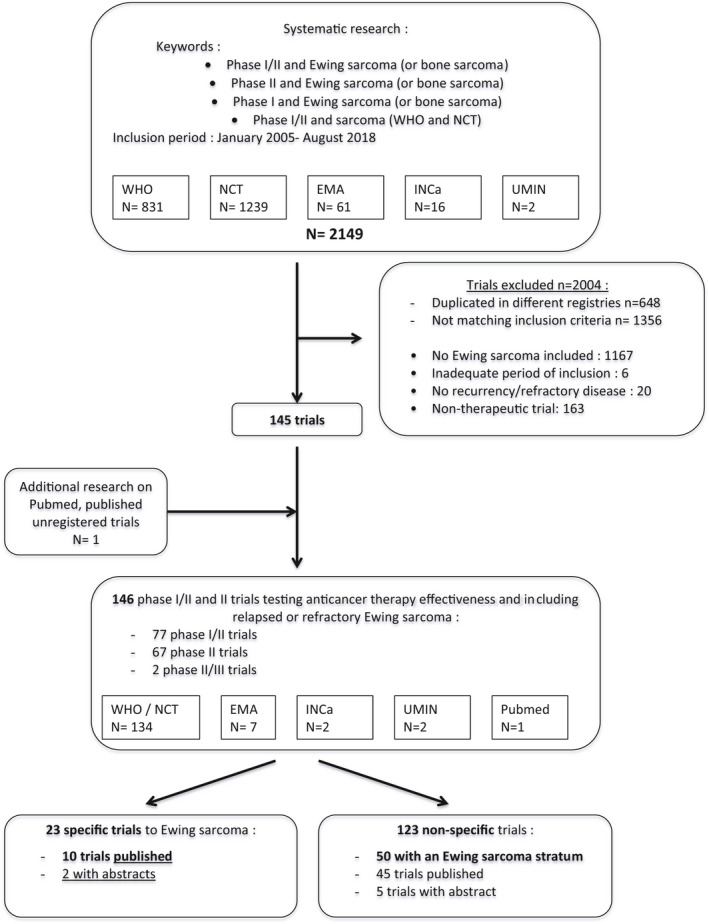
Flow‐chart diagram of Ewing sarcoma phase‐I/II trials selection according to PRISMA recommendations. WHO, World Health Organization; NCT, Clinicaltrials.gov registry; EMA, European Medicines Agency registry; INCa, French Institut National du Cancer; UMIN, Japanese national registry

The initial search identified 2149 trials and 2004 were excluded (Figure 1). One additional published unregistered trial was identified on Pubmed.^7^ A total of 146 clinical trials were included in further analysis. Trial descriptions were extracted from registry websites, 53 publications, ^8^, ^9^, ^10^, ^11^, ^12^, ^13^, ^14^, ^15^, ^16^, ^17^, ^18^, ^19^, ^20^, ^21^, ^22^, ^23^, ^24^, ^25^, ^26^, ^27^, ^28^, ^29^, ^30^, ^31^, ^32^, ^33^, ^34^, ^35^, ^36^, ^37^, ^38^, ^39^, ^40^, ^41^, ^42^, ^43^, ^44^, ^45^, ^46^, ^47^, ^48^, ^49^, ^50^, ^51^, ^52^, ^53^, ^54^, ^55^, ^56^, ^57^, ^58^, ^59^, ^60^, ^61^, ^62^, ^63^ and 9 abstracts (ASCO website n = 6; results section of websites n = 3).

#### 3.1.1 Trial status

Among the 146 selected trials, 77 were phase‐I/II trials, 67 phase‐II trials, and 2 phase‐II/III trials; with 23 ES‐specific trials, 48 with an ES stratum with dedicate analysis, and 75 recruited ES among larger disease inclusion criteria without specific analysis. Trial status was active/recruiting (n = 84), completed/close to recruitment (n = 58), and withdrawn (n = 4; 2 prematurely ended without described reason; 2 withdrawn before enrollment). Sponsorship was academic in 71% (103/146) or pharma driven in 29% (mostly for adult phase‐I/II trials 31/43 trials).

#### 3.1.2 Geographic/temporal distribution

**FIGURE 2:**
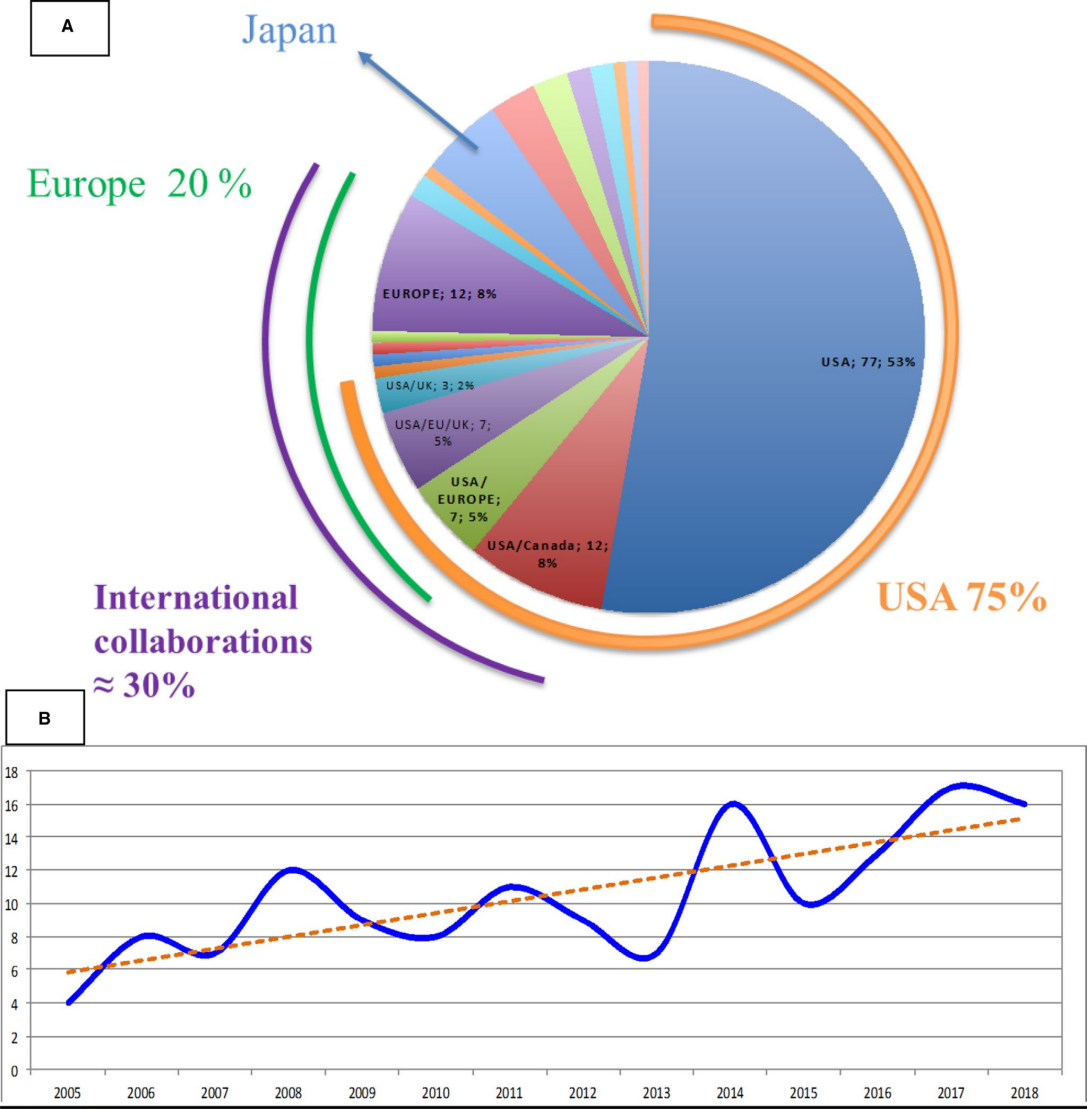
Geographic and temporal distribution of the 146 phase‐II trials in recurrent or refractory Ewing sarcoma. (A) Number of trials opened according to the location. (B) Number of trials opened over time

Trials were mainly multicenter (88%), but usually opened in only one country (77%) and mainly in the USA (53%). A third of the trials were multinational collaborative trials (30%, 44/146) (Figure 2A), and mainly opened after 2008 (77%, 34/44), with a main focused on targeted or immune therapies. The number of trials opening increased since 2014 (8.3 trials/year between 2005 and 2013 vs. 14.4 trials/year between 2014 and 2018) (Figure 2B).

#### 3.1.3 Eligibility criteria

**FIGURE 3:**
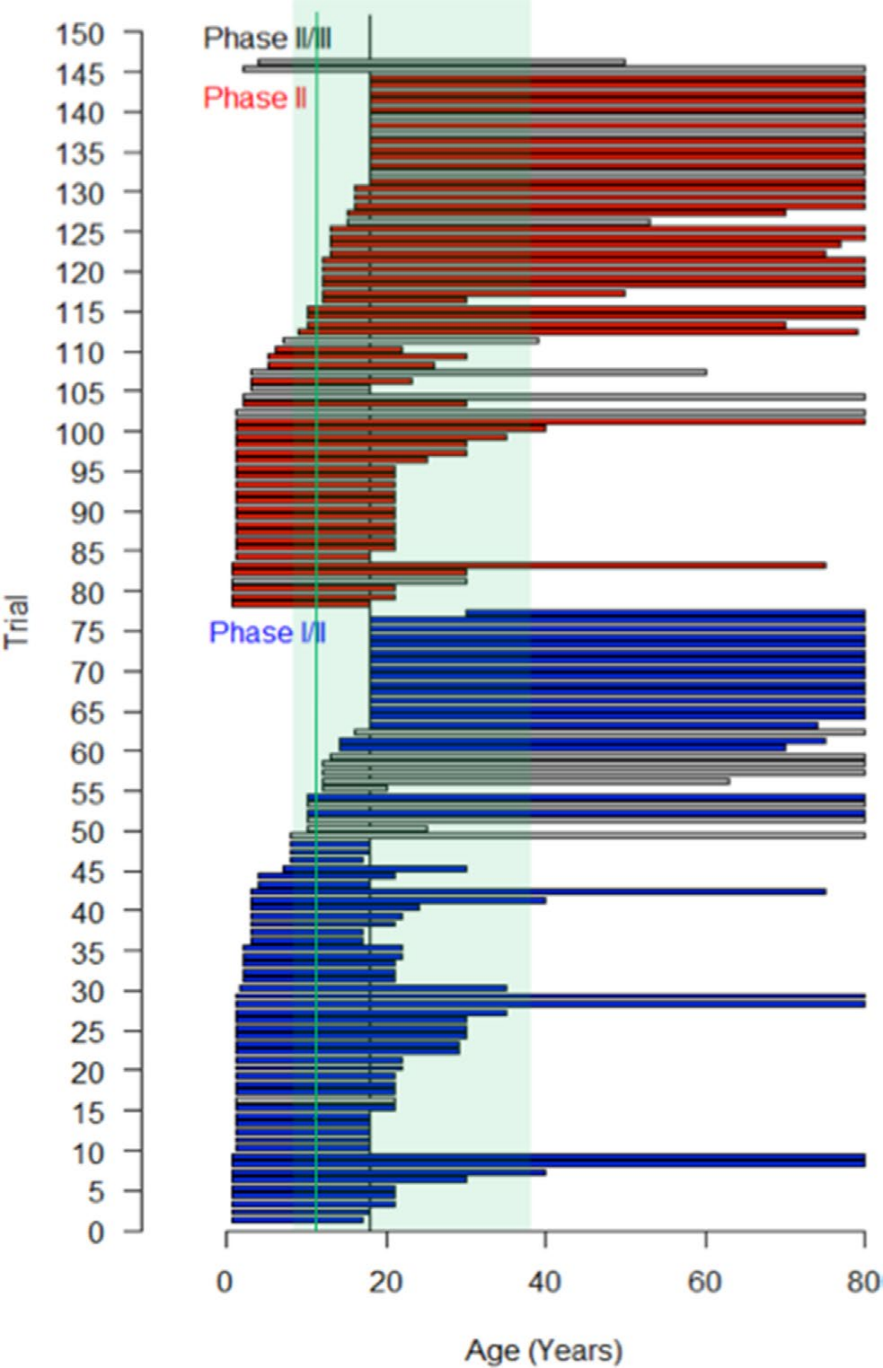
Age eligibility criteria in the 146 selected trials including refractory or recurrent Ewing sarcoma. Each trial is represented by a horizontal bar drawn from the minimal to the maximal age of inclusion. Gray horizontal bars: osteosarcoma specific trials; blue/red horizontal bars: trials with wider eligibility criteria; vertical dark line: 18 years old; vertical green line

Age eligibility criteria allowed both pediatric and adult accrual in 72% of the trials (Figure 3) while 8% were exclusively designed for pediatric (age at inclusion <18 years), and 20% for adult patients only, with an equal distribution over time. Most of the specific pediatric trials tested a drug already available in adult oncology (n = 10/12). Two third of the specific adult trials (n = 10/29) were phase‐I/II testing a first‐in‐human drug. According to the European Euro‐Ewing99 database median age at first relapse is 17.6 years (IQ90%: 7.5–38.4).). Taking this into account, only 12% of the selected trials (n = 17/146) allowed accrual of patients in this age range (8/77 phase‐I/II trials, 7/67 phase‐II trials, and 2/2 phase‐II/III trials), with no differences over time (Figure 3).

One trial included patients in first recurrent/refractory disease only (NCT00516295), while other two were restricted to patients with lung metastasis only (NCT01590069 and NCT00492141). All trials requested measurable disease according to RECIST criteria as inclusion criteria.

#### 3.1.4 Therapeutic intervention tested

**FIGURE 4:**
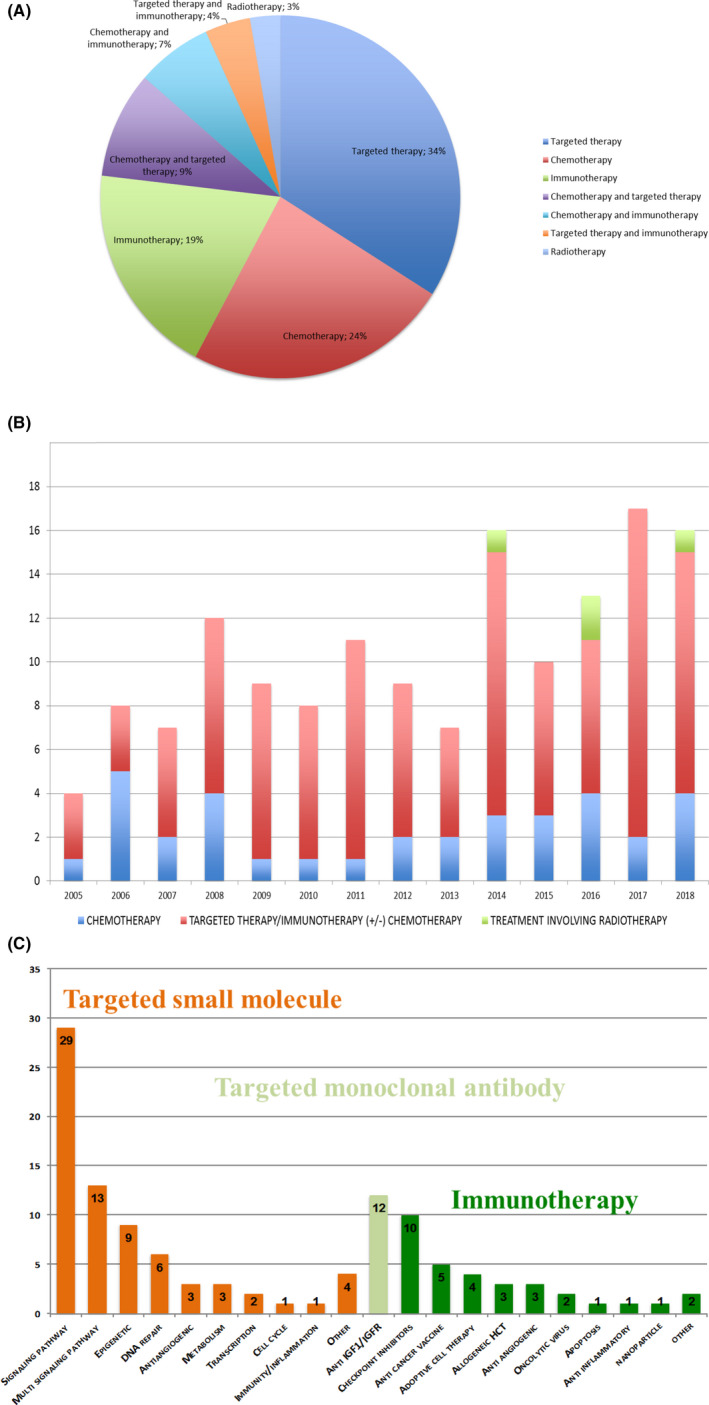
Therapeutic interventions tested in the 146 selected phase‐I/II trials in recurrent or refractory Ewin sarcoma. (A) Therapeutic intervention type. (B) Therapeutic intervention over time between 2005 and 2018. (C) Targeted pathways in trials for targeted therapy drugs (Orange) and immunotherapy drugs (Green). Signaling pathway: Pi3k/Akt/mTOR, Igfr/ir, Raf‐braf, Tgfβ1, Sonic hedgehog, Alk, Errb, Ntrk, and Pak4. Multisignaling pathway: any multi‐tyrosine kinase Inhibitor. Epigenetic: histone deacetylase, histone demethylase‐Inhibitor, and ezh2. DNA repair: parp inhibitors. Antiangiogenic: vegf and fgf/fgfr. Transcription: anti Ews‐fli1. Metabolism: Aldh, Ascorbate, and Statin. Cell cycle: cdk4/cdk6. Immunity/inflammation: Anti‐COX2. Other: molecular‐targeted trials and Xpo‐1. Checkpoint inhibitors: antibody against PD1, PDL1, CTLA4. Adoptive cell therapy: immunotherapy that uses the patient's own T cells after in vitro treatment. Antiangiogenic antibody targeted VEGF. Anti‐inflammatory immunotherapy: IL2 aerosol. 12 years old

Trials evaluated mainly single drugs (90/146, 62%) rather than drug combinations (56/146, 38%) (Figure 4A). The number of trials testing chemotherapy remained stable over time and represents a quarter of the identified trials (n = 34/146), either alone or in combination. From 2007, the number of trials testing targeted and immune therapies increased simultaneously (Figure 4B). Seventy‐one trials (48%) tested targeted therapies, mostly as single agent (n = 50) and 21 combined with chemo‐ (n = 14), immune‐ (n = 6), or radiotherapy (n = 1). The pathways targeted are shown in Figure 4C. We retrieved 44 immunotherapy trials (30%) mostly as single agent (n = 28) and 16 combined with chemo‐ (n = 10), targeted (n = 6), or radiotherapy (n = 1). Immunotherapies targeted mainly the IGF1‐IGFR1 pathway between 2005 and 2012 (12 trials, including 4 ES‐specific trials), checkpoint inhibitors between 2015 and 2017 (10 trials), and anticancer vaccine (n = 5). Two trials included radiation therapy in investigational treatment.

#### 3.1.5 Trials designs

Single‐arm design was the most often used (88%, n = 129/146), both in phase‐I/II (n = 69/77) and phase‐II trials (n = 59/67). Multiple‐arm design was used in 18 trials, with only 7 randomized trials (Figure S1). The 11 non‐randomized multi‐arm trials were phase‐I/II (n = 8) and phase‐II trials (n = 3) and tested parallel arms of the same treatment on different tumor types (basket‐trials n = 5/11), different dosing (n = 3/11), and combination (n = 3/11). The 7/69 randomized phase‐II and II/III trials (10%) compared the investigational drug against placebo (n = 2: Regorafenib NCT02048371 and NCT02389244), several combinations of chemotherapy and/or immune therapy (n = 4: NCT00516295, EudraCT: 2014–000259–99, NCT02511132, and NCT01154452), and different dosing (n = 1, NCT01896505) (Figure S1).

#### 3.1.6 Primary outcomes

Primary efficacy outcomes and measure methods varied greatly between trials (Figure S2). Primary endpoint was mainly RR (n = 117/146, 79%) or progression‐free survival (PFS) (n = 21/146, 14%). Overall survival (OS) was used once (NCT00923351) as histological and metabolic response (EudraCT: 2012–000616–28 phase‐II trial of linsitinib). Tumor response assessment criteria, available in 122 trials, was mainly RECIST criteria^64^ (n = 119 trials, 98%). The median timing for efficacy assessment was of 2 months (range: 1–8 months; 62% between 2 and 3 months) corresponding to a median number of 2 cycles (range 1–4 cycles) with a longer delay for immunotherapies compared to other drugs (median 12 months range: 2–60).

### 3.2 Efficacy results description of the 62 published ES phase‐II trials

#### 3.2.1 Published trial status and design

Globally, 827 patients were accrued (93 patients in phase‐I part of phase‐I/II trials, 234 in phase‐II part of phase‐I/II trials, and 500 in phase‐II trials). A peak of enrollment was observed in 2007–2008 (several anti‐IGF‐1R trials launched in this period) (Figure S3).

On December 2018, 62/146 trials had complete (n = 53) or partial (n = 9 abstracts) published results (Table 1). These trials were mainly multicentric (74%), but rarely multinational (34%). Therapies tested were single agent chemotherapy (n = 12), immunotherapy (n = 15), targeted therapy (n = 15), and 20 combination therapies (32%).

**TABLE 1:** Efficacy results of the 62 fully or partially published early phase trials for recurrent/refractory Ewing sarcoma

Number/ID	Intervention	Age (years)	Number of ES patients	CR	PR	SD	PD	RR (%)	DCR (%)	4‐months PFS (%)	6‐months PFS (%)	Median PFS (months)	6‐months OS (%)	1‐year OS (%)	2‐year OS (%)	Median OS (months)
NCT00474760 +	Figitumumab	12–63	16	1	1	6	5	15	62	.	.	5.6	.	.	.	.
NCT00642941 +	R1507	>2	115	1	10	8	96	10	17	11	9	1.3	60	35	10	7.6
NCT00617890 +	Robatumumab	9–79	100	6	0	23	55	7	35	.	.	.	75	55	16	6.9
NCT00563680	Ganitumab	>16	22	0	1	7	10	6	44	.	.	<2	.	.	.	.
NCT00609141	Cixutumumab	<21	35	0	3	5	27	9	23	.	.	1.5	.	.	.	.
NCT00668148	Cixutumumab	>12	18	0	1	5	13	6	33	27.3	11	1.5	77.8	.	.	6
NCT00560235	Figitumumab	10–25	107	0	15	25	66	14	38	25	2	1.9	60	35	2	8.9
NCT01016015	Temsirolimus + cixutumumab	>16	27	0	4	0	23	15	15	.	.	7.5	.	.	.	16.2
NCT01614795	Cixutumumab + temsirolimus	<30	12	0	0	1	10	0	9	.	.	.	.	.	.	.
NCT02304458^a^	Nivolumab with or without ipilimumab	1–30	10	0	0	0	10	0	0	.	.	.	.	.	.	.
NCT02301039	Pembrolizumab	16–81	13	0	0	2	11	0	15	.	.	.	.	.	.	.
NCT02541604^a^	Atezolizumab	2–29	11	0	0	0	11	0	0	.	.	.	.	.	.	.
NCT00357500	Etoposide, cyclophosphamide + thalidomide, celecoxib	<22	4	0	0	1	0	.	.	8.3	0	3	4	12	0	7
NCT00073983	Gemcitabine + docetaxel	13–77	14	0	2	6	6	14	57	.	.	.	.	.	.	.
UMIN000001037^a^ +	Topotecan and ifosfamide	1–28	6	1	0	2	3	.	.	.	.	.	.	.	.	.
NCT01380275 +	Docetaxel + irinotecan	<30	10	1	2	1	5	30	40	33	33	2.2	.	.	.	.
NCT00807261	Docetaxel and fixed‐dose rate gemcitabine	>16	7	0	1	2	2	.	.	.	.	.	.	.	.	.
NCT01141244 +	Temsirolimus, irinotecan and temozolomide	<22	7	0	1	1	5	.	.	.	.	.	.	.	.	.
NCT02116777	Talazoparib + temozolomide	4–25	10	0	0	2	8	0	20	.	.	.	.	.	.	
NCT00516295^a^	Vincristine+topotecan+cyclophosphamide with bevacizumab	12–20	7	.	.	.	.	.	.	.	.	14.7	.	.	.	.
NCT00331643	Ixabepilone	3–35	16	0	0	1	15	0	6	.	.	<2.2	.	.	.	.
NCT00470275	Cytarabine	<30	10	0	0	1	9	0	10	.	.	<1.5	.	.	.	.
NCT00520936	Pemetrexed	3–23	10	0	0	1	9	0	10	.	.	<1.5	.	.	.	.
NCT00070109	Trabectedin	<21	11	0	0	1	9	0	10	9	9	.	.	.	.	.
EudraCT: 2005‐003254‐10	Oral treosulfan	3–50	21	0	0	1	20	0	5	0	0	1.8	52	2	.	6.4
NCT01222767	Zalypsis^®^ (PM00104)	15–53	17	0	0	4	12	0	25	28.6	.	1.8	.	.	.	.
NCT01610570	Mithramycin	>1	8	0	0	0	8	0	0	.	.	<2	.	.	.	.
NCT00998361	Hematopoietic stem cell transplantation	6–22	10	.	.	.	.	.	.	.	.	.	.	43	.	.
NCT01804634 +	Reduced intensity conditioning and haploidentical BMT	5–26	4	3	1	0	0	.	.	.	.	10	.	.	.	14.6
EudraCT: 2012‐000616‐28^a^	Linsitinib	>18	16	0	0	7	7	0	50	.	.	1.3	.	.	.	7.1
NCT00923351^a^ +	Vaccine and R‐hIL‐7	1–35	21	.	.	.	.	.	.	.	.	.	.	.	.	30
NCT00902044	Her2 chimeric antigen receptor expressing T cells	7–30	1	0	0	0	1	0	0	.	.	.	.	.	.	5
None^a^	Activated haploidentical natural killer cell infusions	5–17	2	0	1	1	0	.	.	.	.	.	.	.	.	.
NCT01241162	Dendritic cell vaccine with or without gemcitabine	<18	2	0	0	0	1	0	0	.	.	.	.	.	.	.
NCT00464620	Dasatinib	>13	17	0	1	0	16	6	6	6	6	1.9	.	.	7	.
NCT02048371^a^ +	Regorafenib	>18	30	0	3	18	7	11	75	73	.	3.6	.	.	.	.
NCT02243605^a^ +	Cabozantinib‐s‐malate	13–74	45	0	9	10	14	27	58	.	24	.	.	.	.	.
NCT01830153	Everolimus	>18	2	0	0	1	1	0	.	.	.	.	.	.	.	.
NCT01286987	Talazoparib	>18	14	0	0	3	9	0	25	.	.	.	.	.	.	.
NCT01583543	Olaparib	>18	12	0	0	4	8	0	33	85	35	5.7	.	.	.	.
NCT02454972^a^	Lurbinectedin	18–74	28	0	4	12	12	14	57	.	.	2.8	.	.	.	.
NCT01962103	Weekly Nab‐paclitaxel	2–17	13	1	1	3	7	17	42	.	.	.	.	.	.	.
NCT01061840 +	Vigil immunotherapy with irinotecan and temozolomide	>2	13	0	1	8	4	8	69	.	.	.	75	73	4	24
NCT00101270	Oxaliplatin + irinotecan	<21	1	0	0	0	1	0	0	.	.	.	.	.	.	.
NCT00321581	Cedinarib	8–18	3	0	1	0	2	.	.	.	.	.	.	.	.	.
NCT00428272	Lexatumumab	2–21	4	0	0	0	4	.	.	.	.	.	.	.	.	.
NCT00776867	Perifosine	<21	1	0	0	0	1	0	.	.	.	.	.	.	.	.
NCT00786669	Bevacizumab and vincristine, irinotecan and temozolomide	<30	2	1	1	0	0	.	.	.	.	.	.	.	.	.
NCT00927966	Figitumumab + everolimus	>18	1	0	1	0	0	.	.	.	.	.	.	.	.	.
NCT00929903	Pazopanib	3–24	3	0	0	0	3	0	.	.	.	.	.	.	.	.
NCT01132911	Vorinostat and bortezomib	<22	2	0	0	0	2	0	.	.	.	.	.	.	.	.
NCT01154816	Alisertib	3–21	5	0	0	0	5	0	.	.	.	.	.	.	.	.
NCT01184274	Pracinostat	<18	4	0	0	1	3	0	.	.	.	.	.	.	.	.
NCT01273090	Imetelstat sodium	3–22	6	0	1	0	5	.	.	.	.	.	.	.	.	.
NCT01353625^a^	Oral CC‐115	>18	10	0	0	2	8	0	20	.	.	.	.	.	.	.
NCT01431534	Ridaforolimus	8–17	3	0	0	0	3	0	.	.	.	.	.	.	.	.
NCT01431547	Dalotuzumab +/− ridaforolimus	3–17	7	0	1	0	5	.	.	.	.	.	.	.	.	.
NCT01453283	Trabectedin	8–16	1	0	0	0	1	0	.	.	.	.	.	.	.	.
NCT01709435	Cabozantinib S‐Malate	4–18	4	0	0	1	3	.	.	.	.	5.2	.	.	.	9.8
NCT01748721	MORAb‐004	3–21	5	0	0	0	4	.	.	.	.	.	.	.	.	.
NCT02171260	Eribulin mesylate	3–17	4	0	1	0	3	.	.	.	.	.	.	.	.	.
NCT01331135	Sirolimus + VP16 + cyclophamide + celecoxib	1–30	3	0	0	0	3	0	0	0%	0	NA	NA	NA		

Note

RR and DCR were not calculated if <10 patients were analyzed. In orange: part I of phase I/II trials.

Abbreviations: +, positive phase‐I/II trial results; RR, response rate = CR+PR; DCR, disease control rate = CR+PR+SD; mAb, monoclonal antibody; Tox, toxicity; PFS, progression‐free survival; OS, overall survival; CT, chemotherapy; Combo, combination therapy; Ped, pediatric trials; HDC, high‐dose chemotherapy; BMT, bone marrow transplantation; MTKI, multi‐tyrosine kinase inhibitor.

^a^ Partially published trials.

Fifty‐seven trials were single‐arm designed (92%). Statistical trial design was mainly two‐stage design (76%). Both the null (H0) and alternative hypothesis (H1) varied widely between trials, with H0 RR of 3%–25% and H1 RR of 20%–50% (median delta H1–H0 of 20%) and H0 4 months or 6 months PFS of 10%–25% and H1 4 months or 6 months of 25%–50% (median delta of 20%).

#### 3.2.2 Published trial efficacy results

**FIGURE 5:**
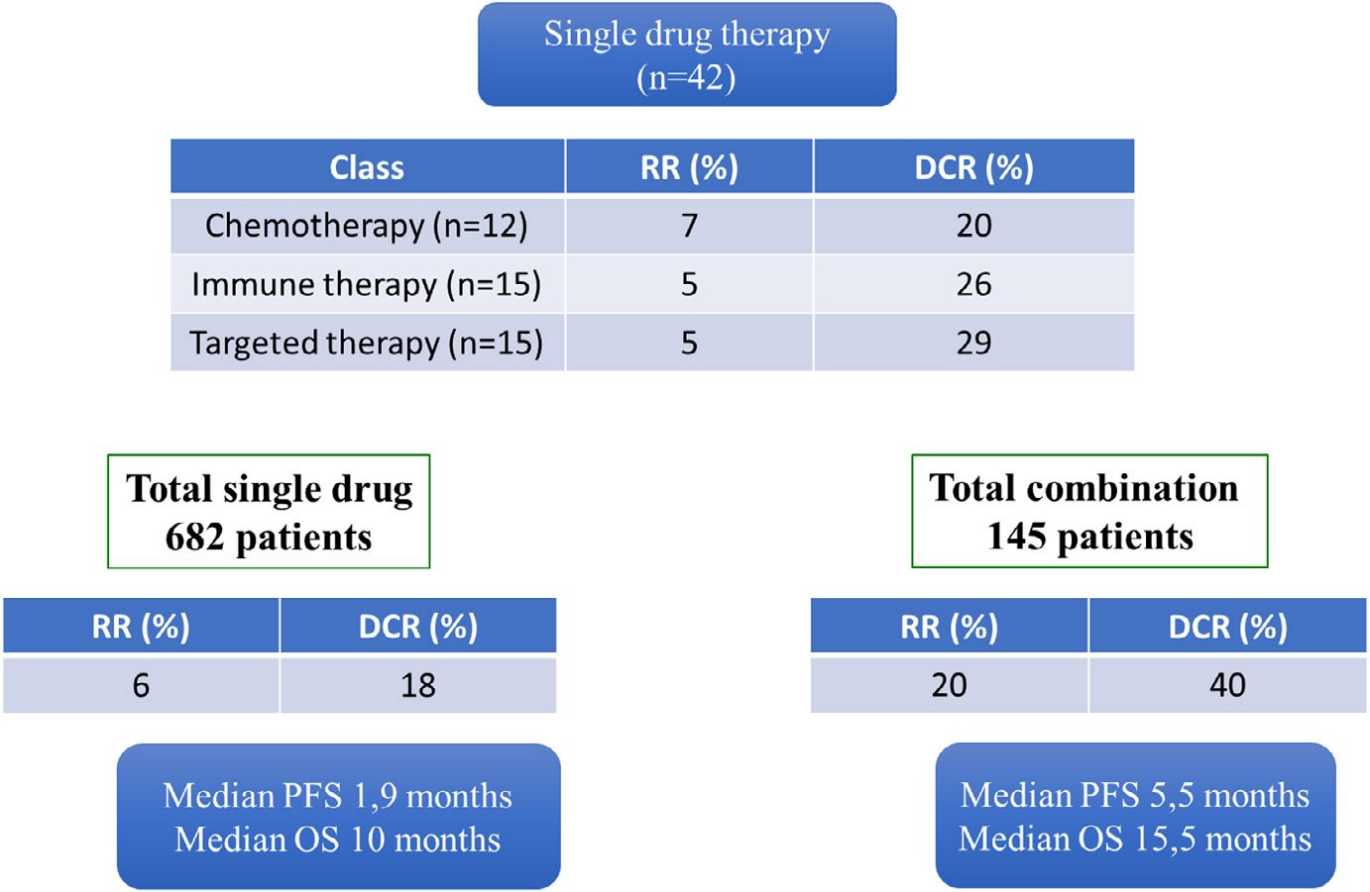
Description of phase‐I/II response rate in the 62 published trials according to pharmaceutical class and single drug versus combination

Among the 758/827 patients (92%) evaluable for response and/or survival assessment, RR was poor (10%) with 13 CR (1.7%) and 63 PR (8.3%). Thirty trials (48%) had at least one objective response (CR or PR). SD was the best response in 186/758 (25%) with a DCR of 35% after 2 months. Two third of the patients experienced PD at first efficacy assessment (Figure 5). The median PFS was of 1.9 months (range 1.3–14.7 months). Only three trials showed a median PFS >6 months ^12^, ^32^, ^63^ (NCT00516295, NCT01016015, and NCT01804634). The median OS was of 7.6 months (range 5–30 months). Three trials had median OS >15 months, all of them tested immunotherapies ^49^, ^63^ (NCT00923351, NCT01061840, and NCT01016015) (Table 1; Table S1).

No difference of RR and survival was observed among the different therapeutic interventions as single agent (chemo, targeted, and immune therapies, Figure 5). Combination therapies, whatever their types tend to have better outcome than single agents. Median RR, DCR, PFS, and OS in single agent trial compared to combination therapy was of 6% versus 18%, 20% versus 40%, 1.9 versus 5.5 months, and 10 versus 15.5 months, respectively (Figure 5).

No RR and survival improvement was observed throughout time. We did not find correlation between RR and survival.

At first evaluation, response evaluation was PD in 14 trials (23%), and SD in 18 trials (29%). Eleven trials (18%) were considered positive (Table S1) with median RR, DCR, PFS, and OS of 15% (range 7%–30%), 51% (17%–75%), 4.5 months (1.3–10 months), and 16.6 months (6.9–30 months), respectively, (three combined chemotherapies, three anti‐IGFR, three immunotherapies, and two MTKI). The 51 alleged negative trials had a median RR, DCR, PFS, and OS were of 3% (range 0%–17%), 19% (range 0%–44%), 3 months (range 1.7–14 months) and 7.1 months (range 5–16 months), respectively.

## 4 DISCUSSION

Life expectancy of patients with recurrence ES has not changed during the last decades and no targeted/immune therapy is routinely used.^65^ Along with an improvement in ES biology understanding and target/drug discovery, appropriate phase‐II trials designs are needed for the development of appropriate and successful phase‐III trials, and improvement of care and survival in such rare diseases.

This review performed with a PRISMA methodology retrieved 146 phase‐II trials with ES accrual in the last 13 years (2005–2018), and highlights some directions that might be useful to improve the design of future phase‐II trials in recurrent/refractory ES.

Our study cover a recent large period (2005–2018) compared to previous literature on the subject (1990–2010),^1^ with a maximized exhaustiveness facilitated by the systemic therapeutic clinical trial registration implemented from 2005 ^66^ and our search method with wide screening criteria on various international registries and publication sources. The inclusion of all trials regardless of the enrollment or publication status, also allowed the analysis of spatial and temporal trends in phase‐I/II trial designs over the last 13 years with minimal publication bias. These strengths of our analysis are balanced by the heterogeneity of available data in unpublished and nonspecific ES trials.

The number of phase‐II trials either accruing or specifically designed for refractory/recurrent ES patients increased compared to a previous review (1990–2010)^1^ and during the current study period (2005–2018), mainly since 2007. This might reflect the better pediatric and adult bone sarcoma expert national/international collaborations,^5^, ^65^, ^67^ as 71% of trials had academic sponsorship. This greater ES phase‐II trial availability was partially attributable to the increase number of trials testing targeted and immune therapies, reflecting increased new drug availability.

However, few ES patients were accrued in the phase‐II trials (827 included in published clinical trials over 13 years, corresponding to 64 patients per year), representing less than 10% of the European and USA population theoretically eligible to participate to such phase‐II trials (with European/USA population estimated to one billion, ES incidence of 2 per million inhabitants, and ES recurrence rate of 30%, around 700 patients per year might suffer refractory/recurrent ES). Several factors might explain trial under‐recruitment.

The geographic availability/accessibility of ES phase‐II trial was highly unequal worldwide. Although usually multicenter, half of the trials were only open in the USA, and only 30% were international collaborative trials. Age range of ES recurrence, in‐between pediatric and adult populations, may have slowed patient's referral to phase II trials. Patient referral for trial remains a challenge, despite several initiative supported by the health care system at national and cross country level,^68^, ^69^ as in the adolescent and young adult (AYA) population,^70^, ^71^ where the perceived benedict of early phase trial participation differing between clinicians, might limit the offering of such trials. It is likely that some of the trials in our report may have included patients with sarcomas with alternative transcripts to the usual EWS‐FLi1 (e.g., CIC or BCOR) instead of the EWSR1 translocations. Future trials may require ES to be more precisely defined by its specific translocation.

Age inclusion criteria, at the opposite to other AJA cancers,^70^, ^71^ often allowed both pediatric and adult population in ES phase‐II trials (72% of joint phase‐II and phase‐II/III trials). AYA collaborative groups in collaboration with bone sarcoma groups at national (e.g., InterSARC group in France) and international level (e.g., EuroEWING Consortium, EEC) might have favor these joint pediatric/adult trials.^3^, ^65^, ^67^, ^72^ However, only 12% of the studies trial cover the 90% age range of first ES recurrence (7.9–38.4 years).^73^, ^74^ With a median age of 17.6 years at first recurrence, an 18‐year age limit either in pediatric or adult initiated trials, excluded half of the ES population from ES phase‐II trials. In addition, adult phase‐I/II trials testing a first‐in‐human drug did not allow adolescent accrual and pediatric phase‐I/II trials started late after the adult development (14 specific pediatric phase‐II trials set‐up while the drug was already tested in adults). Joint adolescent/adult early phase trial with a lower age limit of 12 years old, would allow covering 84% of first ES recurrences, giving early access to new drugs to adolescents, and inform the pediatric development. This strategy, based on disease epidemiology rather than age, is supported by the multistakeholder ACCELERATE FAIR trial initiative,^75^ and regulatory authorities from EMA and FDA (U.S. Food and Drug Administration).^72^, ^75^, ^76^ An adolescent or sarcoma cohort in basket trial could also be discussed.

Requirement of measurable disease according to RECIST criteria ^64^ (extra‐osseous lesion of more than one centimeter) as inclusion criteria in most trials, would have theoretically excluded ES patients at first recurrence with non‐measurable but evaluable disease such as patient with bone or bone marrow metastasis only or multiple pulmonary micro‐nodules. This could be circumvented if, instead of RR as primary endpoint (80% of ES phase‐II trials), PFS would have been used (only 16% trials). This choice imposed by RR as primary endpoint (82% of ES phase‐II trials) is not needed if PFS is used as primary endpoint. Indeed, progression can be evaluated even in nonmeasurable disease either evaluable (bone, bone marrow, and pulmonary micro nodules) or not (minimal residual disease). The definition of SD varied between studies. In some trials, confirmation of SD with a second set of imaging is required, while in others, patients who do not progress on the first imaging are counted as SD even if they progress at second set of imaging. Increasing evidences suggests that RR according to RECIST criteria is not an ES survival surrogate either at diagnosis or relapses.^77^, ^78^, ^79^, ^80^ From 2008, bone experts have recommended to use PFS as a more efficient end‐point in recurrent bone sarcoma phase‐II studies,^81^, ^82^, ^83^, ^84^ without clear translation after this date yet (6/31 trials with PFS criteria before 2009 and 20/116 trials between 2009 and 2018). Moreover, PFS as primary endpoint would require longer evaluation time and has not been standardized.

The statistical design might influence the reliability of the efficacy results of trials. Indeed, most trials had single‐arm design in ES phase‐II trial (88%) or non‐randomized multi‐arm trials (7%), which require appropriate H0 hypothesis^85^ and a clear idea of what would be a success (H1 hypothesis). The lack of historical controls in term of RR and PFS in the highly heterogeneous ES phase‐II trial population (refractory or recurrent diseases, first or subsequent recurrences, local vs. metastatic diseases, and bulky vs. minimal residual disease), probably led to the observed heterogeneity in the H0 hypothesis and the overlap range of H0 and H1 hypotheses between different trials. Consequently, different trials were considered either positive or negative with the same observed RR or PFS. For example, three trials NCT01962103, NCT01016015, and NCT00073983 showed better RR, (respectively, 17, 15, and 14%) than six trials considered as positive. As 70% of trials were academic, ES or bone expert clinicians have great responsibility in the future to design better clinical trials and involve patient association and industrials.

These observations support randomized ES phase‐II trial designs, as does the absence of standardized treatment in refractory/recurrent ES. In randomized trial against placebo, blinded randomization might avoid bias of progression overestimation in the control arm. The design of cross‐over trial at progression, makes the phase‐II randomized approach against placebo more acceptable by clinicians, patients, and ethical committees (e.g., regorafenib trials).^42^, ^86^ However, crossover design of a new agent might compromise the ability to assess clinical benefit on survival. The lack of standard second line chemotherapy for refractory/recurrent ES may explain the rarity of randomized trial against second line chemotherapy and the stable number of single‐arm phase‐II trials testing chemotherapeutic agents over the years. The recruiting rEECUr trial is trying to answer the question of the standard chemotherapy regimen in recurrent/refractory ES with a randomized phase‐II trial with multi‐arm/multi‐stage (MAMS) design ^79^ (EudraCT number: 2014–000259–99). However, randomized trial designs require larger trial sample size than single‐arm trials, which might translate in longer accrual period.

The description of the scientific rationale leading to the trial was well described in published trials, and rather difficult to find in unpublished trials (17/87, 20%). Only seven published trials included preclinical evaluation of the drug in sarcomas (e.g., PARP inhibitors). Redundancy of some trials suggests a drug development in ES rather driven by pharma development policy than biological rational. For example, 10 separate trials tested anti‐IGF1/IGFR inhibitors between 2008 and 2010, based on biological rational and some spectacular early responses.^8^, ^10^, ^15^, ^25^, ^29^, ^59^, ^61^, ^62^, ^63^ However, as concomitant anti‐IGF1/IGFR inhibitor development in adult cancers was stopped for futility the development was also stopped in ES, without further research for efficacy biomarkers resistance mechanisms to the drugs. Ten separate trials testing checkpoint inhibitors between 2015 and 2017, had no clear biological rational in ES.^36^, ^39^, ^43^ International efforts are being made to strengthen biology rational behind early clinical trial in ES,^67^ preclinical drug testing with more reliable in vivo models (e.g., ITCC‐P4 Program) ^87^ and to rationalized drug development in the small pediatric population groups ^3^, ^65^, ^68^(e.g., forum of ACCELERATE ^75^). This should help the emergence of new drugs targeting ES oncogenesis and microenvironment to improve patient survival,^3^, ^4^, ^87^ and hopefully more successful trials.

Overall, efficacy results in the 62 published ES phase II trials were disappointing. The objective RR was only of 10%, lower than in previous review where less targeted and immune therapies were tested^1^ (55% vs. 70% in our study). The highest RR, DCR, PFS, and OS were achieved in trials using combination therapies,^12^, ^13^, ^32^, ^37^, ^49^, ^51^, ^63^ which correlated with single‐center experiences and retrospective studies in countries with difficult access to international trials showing RR>30% for ES recurrent/refractory patients,^88^, ^89^, ^90^ but none compared in a randomized way the efficacy of combination versus single drug. These combination trials are more complicated to design because the rational of the starting dose is unclear and the determination of the relationship between toxicity and doses of multiple drugs remains unpredictable.^91^ In many cases, toxicity does not overlap between cytotoxic drugs and targeted agents and these combinations can theoretically modulate chemo‐resistance pathways without increasing toxicity (except therapies affecting DNA repair). These multidrug trials combining chemotherapy with another agent seem to represent a promising strategy for future phase‐II trials in ES.

In conclusion, the analysis of phase‐I/II trials opened in the last 13 years for recurrent/refractory ES are disappointing. The heterogeneity in phase‐II trial methodology and the lack of historical data highlight the need to optimize trial design. Earlier access to new drugs in ES could be accelerated by joint adult/adolescent basket phase‐I/II trials. International, randomized phase‐II trials, trans‐age, with measurable and/or evaluable disease at study entry, with PFS as standardized primary endpoints should be promoted to define standard treatment, and should be based on a better understanding of ES biology (Figure S4). All this strategy requires better preclinical testing, and collaboration between scientists, medical/pediatric oncologists, health authorities, and pharma industry.

## CONFLICT OF INTEREST

The authors declare no potential conflict of interest.

## AUTHOR CONTRIBUTIONS

Arthur Felix contributed to the design and implementation of the research, performed the data extraction, to the analysis of the results, and to the writing and correction of the manuscript. Pablo Berlanga contributed to the design and implementation of the research, performed the data extraction and the analysis of the results, and corrected the manuscript. Maud Toulmonde contributed to the implementation of the research and the analysis of the results and corrected the manuscript. Judith Landman‐Parker contributed to the implementation of the research and the analysis of the results and corrected the manuscript. Sarah Dumont contributed to the implementation of the research and the analysis of the results and corrected the manuscript. Gilles Vassal contributed to the implementation of the research and the analysis of the results and corrected the manuscript. Marie‐Cécile Le Deley contributed to the implementation of the research and the analysis of the results and corrected the manuscript. Nathalie Gaspar contributed to the design and implementation of the research, to the analysis of the results, and to the writing and correction of the manuscript.

## ETHICAL APPROVAL

We obtained ethical approval for this research by Paris René Descartes university ethical committee.

## Supporting information

Fig S1‐4

Table S1

Table S2

Fig S4
