# Supplementary material for: Systematic review of phase‐I/II trials enrolling refractory and recurrent Ewing sarcoma: Actual knowledge and future directions to optimize the research
Source: Cancer Med. 2021 Jan 15;10(5):1589–604. doi: 10.1002/cam4.3712 (PMC7940237; doi:10.1002/cam4.3712)
Supplement: Supplementary file 1 — Fig S1‐4 [file CAM4-10-1589-s004.pdf]

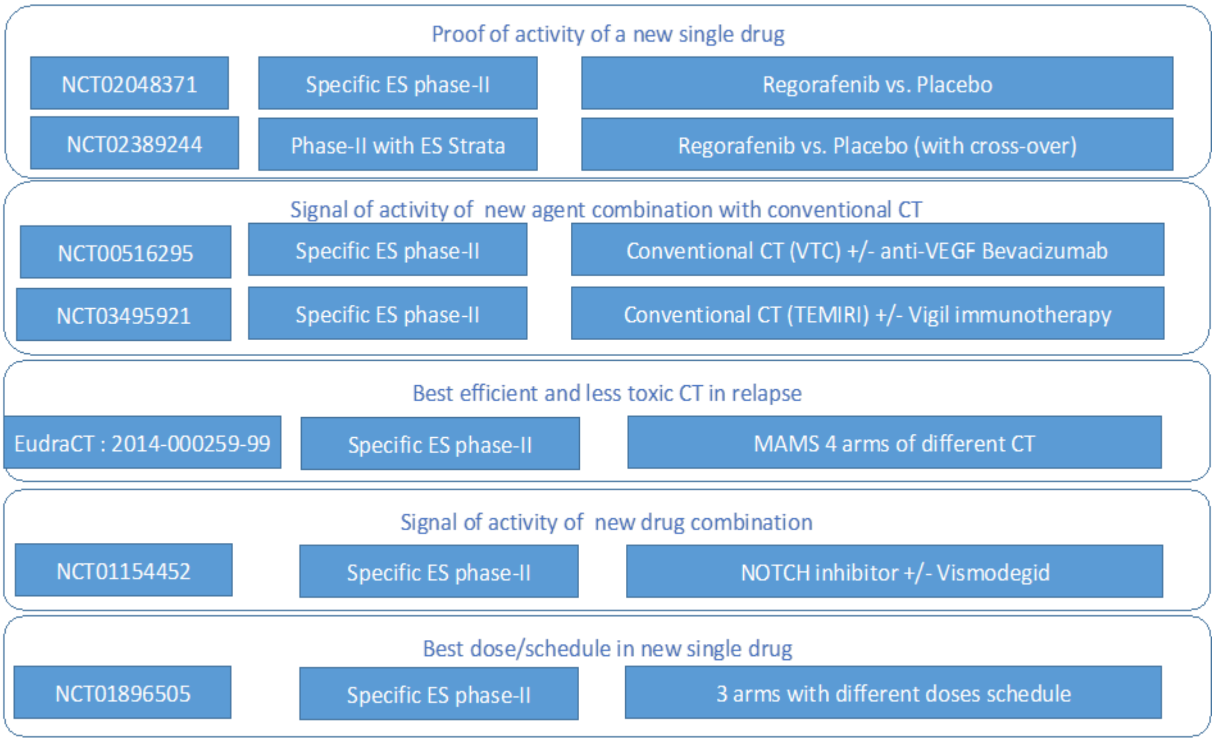

Figure S1: Multi arm randomized phase–II trial design in Ewing sarcoma between 2005-2018 (7 trials). *CT: Chemotherapy. VTC: Vincristine, Topotecan, Cyclophosphamide. TEMIRI: Temozolomide + Irinotecan. MAMS: Multi-Arm Multi-Stage.*

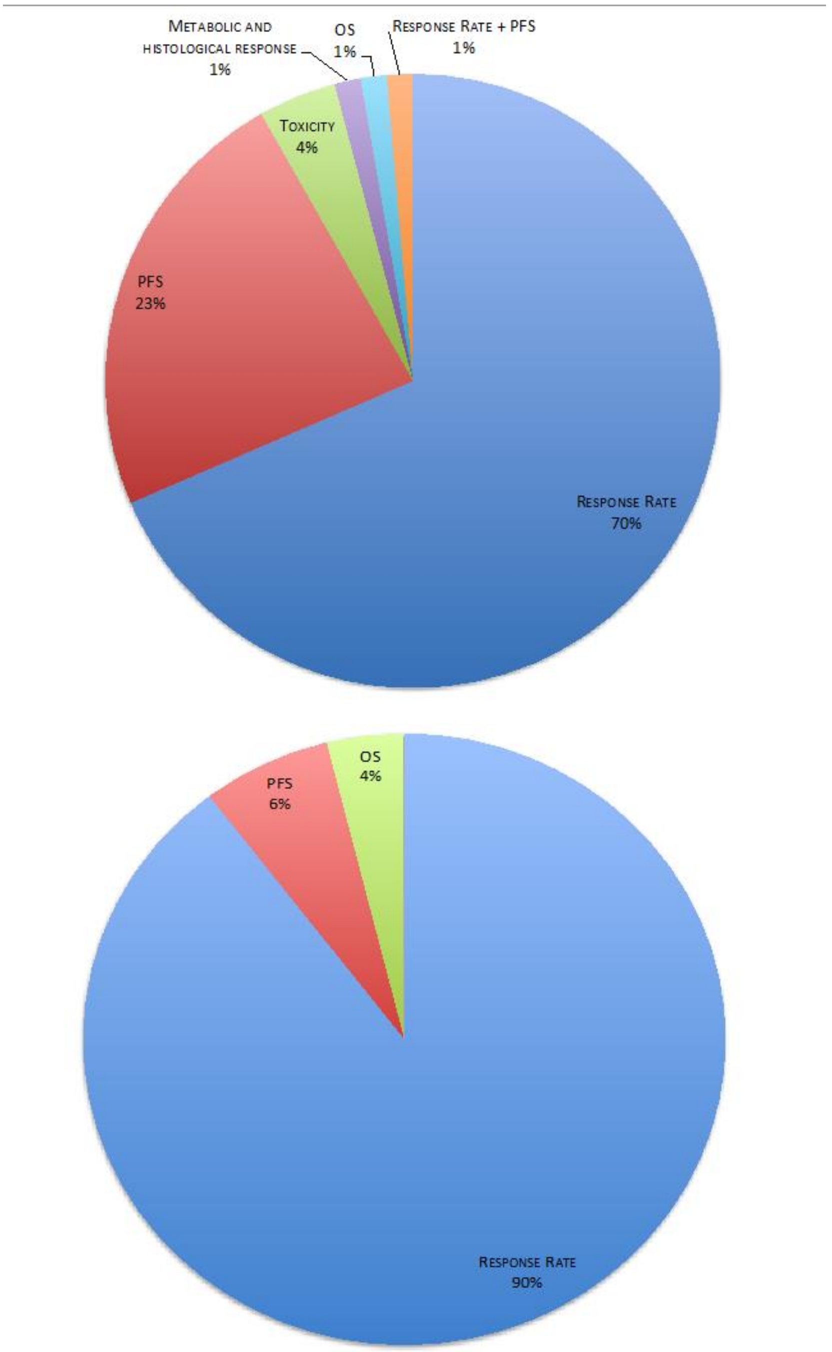

<u>Figure S2:</u> Primary efficacy endpoints in Ewing sarcoma phase-II trials between 2005 and 2018 (*A) In the 67 phase-II and 2 phase-II-III trials and (B) In the phase-II part of the 77 phase-I/II trials.*

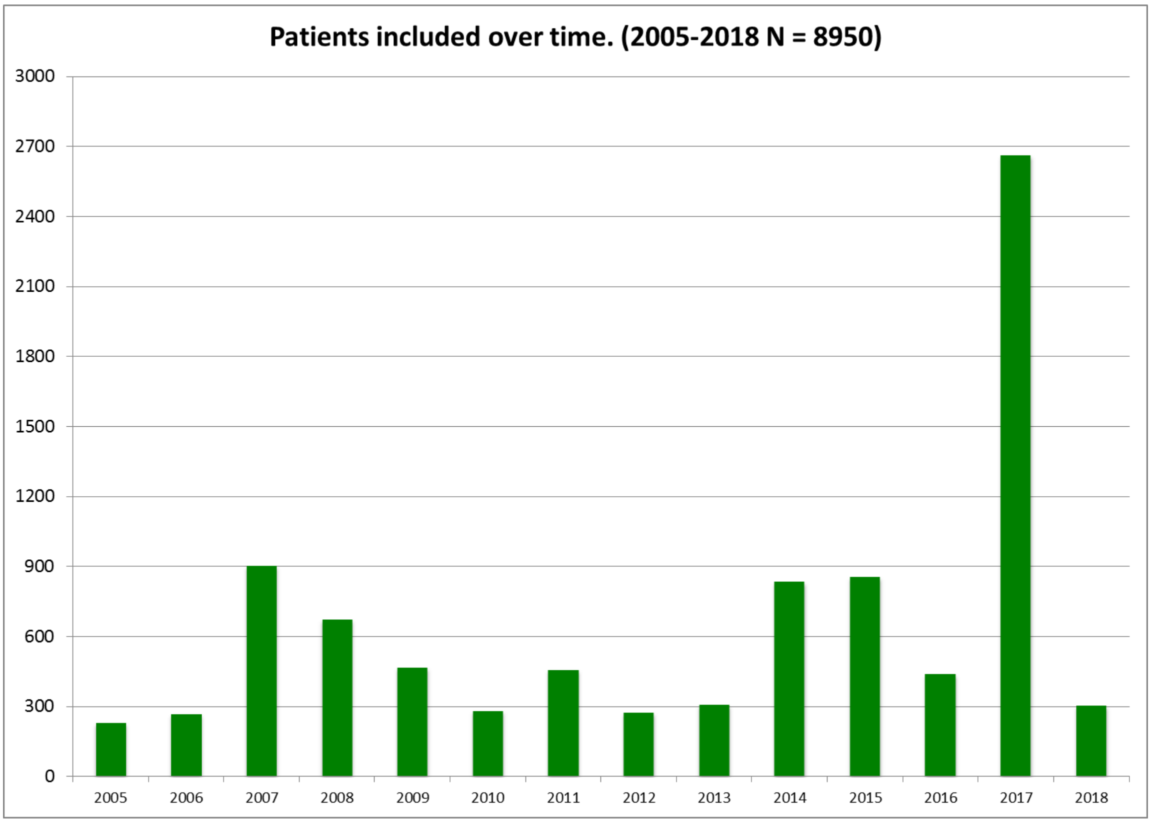

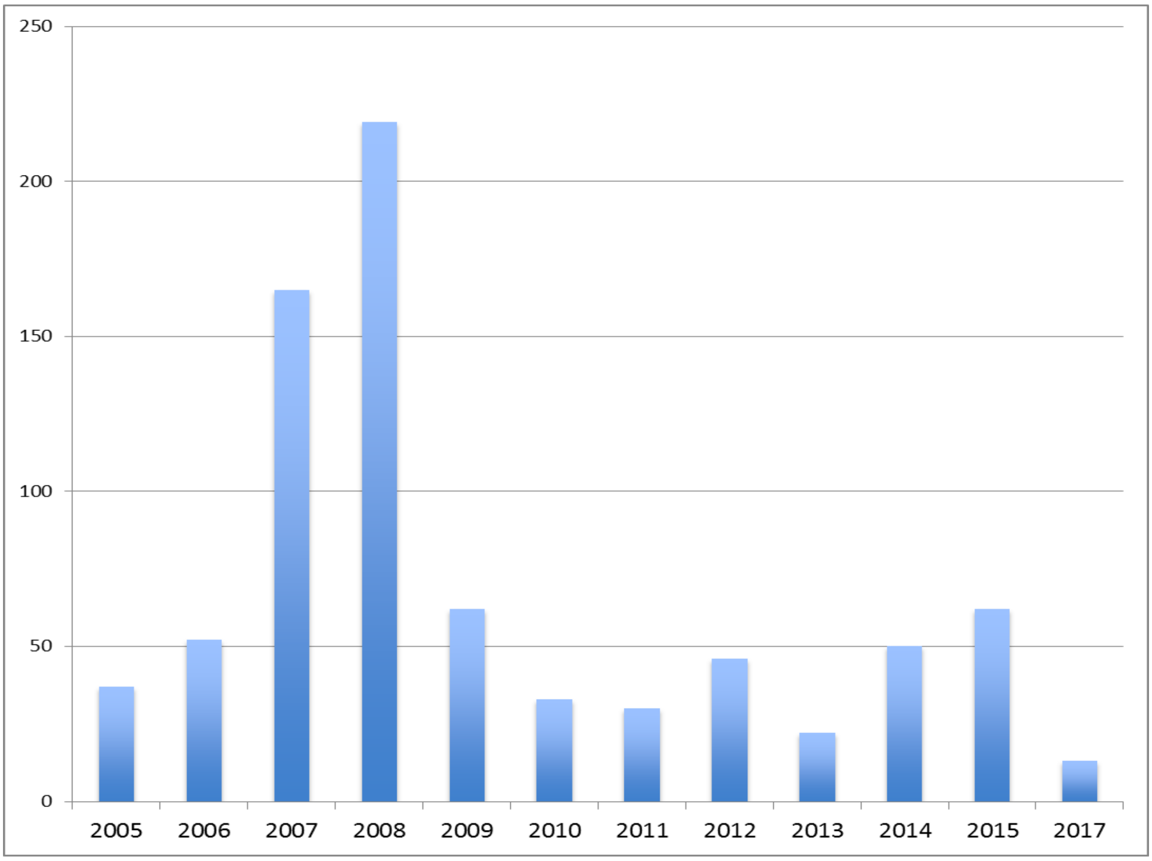

<u>Figure S3:</u> Patients accrued in Ewing Sarcoma Phase-II trials over time between 2005 and 2018, classified by the year of trial opening. A. *In the 146 trials B. ES patients accrued in the 62 published early-phase trials.*

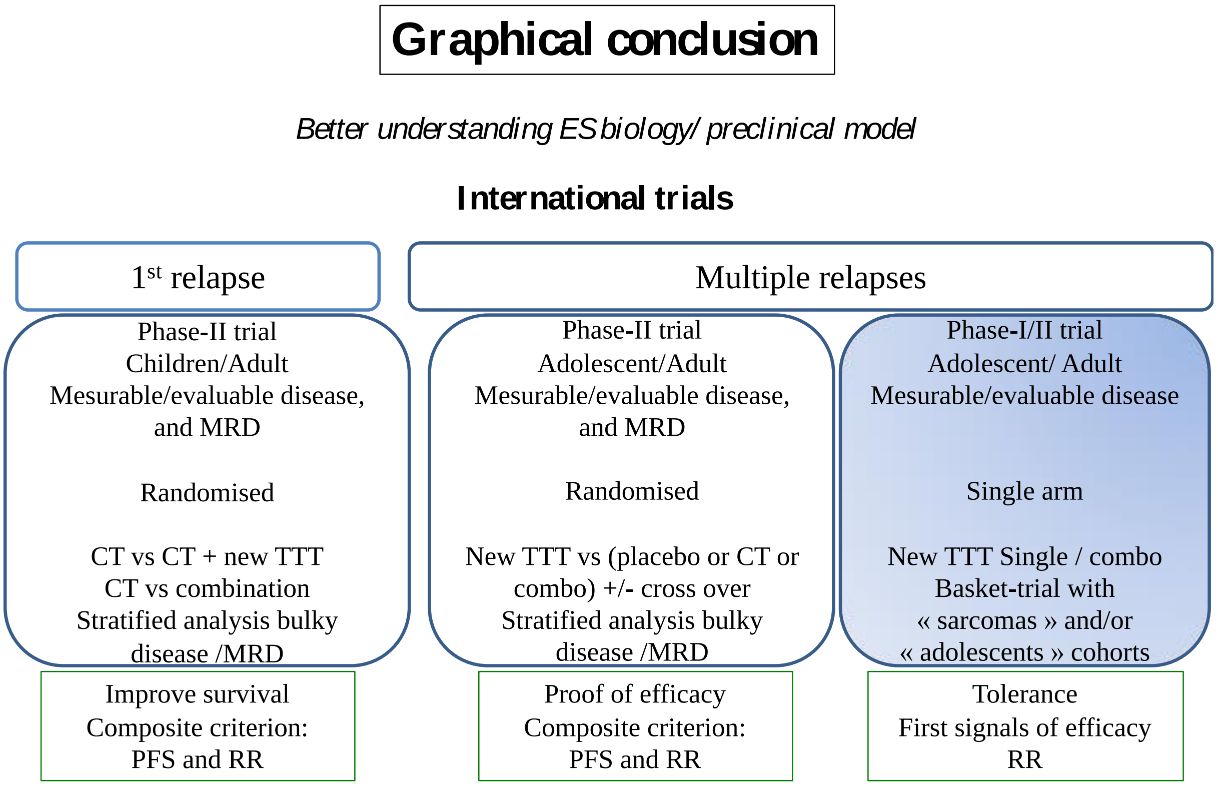

Figure S4: Graphical conclusion and proposals for future phase-I/II trials enrolling ES recurrent or refractory patients. *CT: Chemotherapy, MRD: minimal residual disease, TTT: treatment, Combo: combination.*
