# Supplementary material for: Systematic review of phase‐I/II trials enrolling refractory and recurrent Ewing sarcoma: Actual knowledge and future directions to optimize the research
Source: Cancer Med. 2021 Jan 15;10(5):1589–604. doi: 10.1002/cam4.3712 (PMC7940237; doi:10.1002/cam4.3712)
Supplement: Supplementary file 3 — Table S2 [file CAM4-10-1589-s002.docx]

**Table S2: Description of 146 phase-I/II trials including refractory/recurrent ES between 2005-2018**
