# Supplementary material for: Systematic review of phase‐I/II trials enrolling refractory and recurrent Ewing sarcoma: Actual knowledge and future directions to optimize the research
Source: Cancer Med. 2021 Jan 15;10(5):1589–604. doi: 10.1002/cam4.3712 (PMC7940237; doi:10.1002/cam4.3712)
Supplement: Supplementary file 4 — Fig S4 [file CAM4-10-1589-s003.pdf]

Graphical conclusion

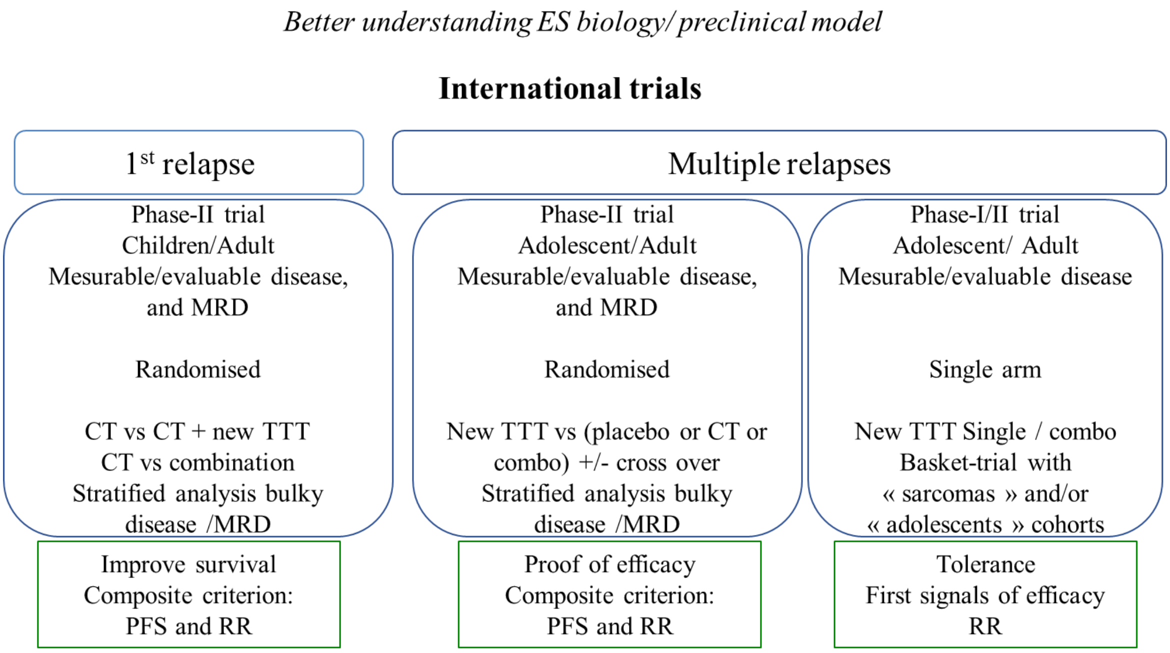

<u>Figure S4:</u> Graphical conclusion and proposals for future phase-I/II trials enrolling ES recurrent or refractory patients. *CT: Chemotherapy, MRD: minimal residual disease, TTT: treatment, Combo: combination.*
